# Editorial: Indigenous Research of Personality From Perspectives of Globalization and Glocalization

**DOI:** 10.3389/fpsyg.2022.864494

**Published:** 2022-04-26

**Authors:** Weiqiao Fan, Frederick T. L. Leong, Sumaya Laher, Mingjie Zhou, Kexin Wang

**Affiliations:** ^1^Research Institute for International and Comparative Education and Department of Psychology, Shanghai Normal University, Shanghai, China; ^2^Retired, East Lansing, MI, United States; ^3^Faculty of Humanities, School of Human and Community Development, University of the Witwatersrand, Johannesburg, South Africa; ^4^Institute of Psychology, Chinese Academy of Sciences (CAS), Beijing, China; ^5^College of Media and International Culture, Zhejiang University, Hangzhou, China

**Keywords:** indigenous research, personality, globalization, glocalization, self, relatedness

A long-term challenge in studying personality has been to strike a balance between seeking the universality of structure and describing the rich variety in personality due to cultural and background differences (Cheung et al., 2011). In the past, personality research has been dominated by Western-based theories, and self-construction has been the major emphasis (Cheek and Cheek, 2018). However, due to the neglect of the cultural particularity of personality (e.g., interpersonal relatedness), Western-based theories have been challenged in theory and practice when used in non-western contexts (e.g., Fan et al., 2011; Thalmayer et al., 2020). Accordingly, beyond those traditional Western-based studies, non-Western-based personality research focusing on the indigenously addressing personality in non-Western contexts started since the 1970s (McAdams and Pals, 2006). There are two basic types of indigenous research in non-Western personality. If we regard the imported-etic research on the personality of mainstream Western psychology in non-Western contexts as a manifestation of globalization, then understanding the construct of Western-based personality with non-Western thinking can be regarded as glocalization in indigenous research of personality. This involves the transport and test paradigm with the usual path from Western to non-Western cultures. The other type of indigenous personality research is, beyond those Western-based personality constructs, to construct and explore the personality embedded in specific non-Western cultural contexts. This usually involves a bottom up approach when local observations give rise to theory building and testing. Over the past five decades, a substantial amount of indigenous research has been devoted to addressing personality in non-Western, for example, Chinese and South African communities, and specifically investigating how Western-based personality constructs, or indigenous personality constructs in explaining ones' behaviors in specific non-Western settings (e.g., Cheung et al., 2011). Furthermore, with the worldwide interactions among various cultures (e.g., between Eastern and Western cultures), some indigenous personality constructs proposed in non-Western backgrounds have also been paid attention to in Western backgrounds (e.g., Lin and Church, 2004; Thalmayer et al., 2020). This may be considered to be another type of the glocalization from non-West to West in indigenous personality research. However, neither the abovementioned two types of indigenous personality research nor the glocalization research of non-Western personality in Western contexts has been adequately represented in the mainstream international personality research community. Accordingly, as we hoped in the proposal, the articles in this special issue explored some important topics in relation to theory and application of personality constructs from perspectives of globalization and glocalization.

Based on a review of the broad dualistic model of self and relatedness supported in both western and eastern cultural settings, Fan, Li, Leong, et al. reconstructed a new two polarities personality model including not only self and relatedness but also the independent and interdependent functions. They argued that, in terms of the cultural-relevant feature, both self and relatedness and their specific aspects may be variously highlighted in different cultural settings by either independent or interdependent function. This updated model may function better when used in cross-cultural studies since some cultures are individualistic and other cultures are collectivistic. Specifically, in three empirical studies, the validities of interpersonal relatedness personality have been empirically examined in a sample of Chinese entrepreneurs (Zhou, Huang et al.) and two samples of Chinese adolescents (Fan, Li, and Chen et al.; Li). In contrast, with a perspective of globalization, Zhang highlighted the universal natures of personality in light of a review on personality-based intellectual style models including the Jungian personality and Holland hexagonal personality.

In terms of how to reflect and measure indigenous personalities in specific contexts, two articles are published in this special issue. Zhou, Mu et al. examined the reliability and validity of the Short Forms of the Cross-Cultural (Chinese) Personality Assessment Inventory, which was originally developed in a Chinese cultural setting with a combined emic–etic approach (Cheung et al., 2011) and reflected a dualistic personality model including both intrapsychic and interpsychic dimensions of personality traits. Thalmayer et al. described the creation of a personality inventory tailored to a specific population—Khoekhoegowab speakers in Namibia—and assesses its psychometric properties and predictive ability for physical and mental health, religious practice and attitudes, and income.

The final three articles addressed western-derived personality in Chinese cultural contexts from a perspective of glocalization. Hence those personality constructs, originally derived in western-based individualist backgrounds, may have culturally-relevant understanding and predictive ability in Eastern backgrounds. Liu et al. examined how dark triad traits contribute to eudaimonic well-being. Yue et al. explored the relationships among self-appraisals, reflected appraisals and peers' actual appraisals of the Big Five Personality. Finally, Peng et al. used the systematic review method to identify 25 short versions of the ZTPI and used these to investigate the structural validity and internal consistency of three short forms of the Zimbardo Time Perspective Inventory in Chinese samples.

To sum up, within the context and current era of globalization and glocalization, ten articles in this Research Topic provide a showcase for recent advances in indigenous research of personality. We believe the Research Topic in this special issue contributes to a more inclusive understanding of personality and will inspire new thinking for personality research from both perspectives of globalization and glocalization under either western or eastern cultural settings.

## Author Contributions

All authors listed have made a substantial, direct, and intellectual contribution to the work and approved it for publication.

## Funding

This work was funded by the National Social Science Fund of China-Education (Grant No. BIA210175).

## Conflict of Interest

The authors declare that the research was conducted in the absence of any commercial or financial relationships that could be construed as a potential conflict of interest.

## Publisher's Note

All claims expressed in this article are solely those of the authors and do not necessarily represent those of their affiliated organizations, or those of the publisher, the editors and the reviewers. Any product that may be evaluated in this article, or claim that may be made by its manufacturer, is not guaranteed or endorsed by the publisher.
